# The effect of short stories on secondary school students’ reading comprehension skills and attitudes in Northwest Ethiopia

**DOI:** 10.1371/journal.pone.0350250

**Published:** 2026-06-01

**Authors:** Yewulsew Godie Gela, Birtukan Gizachew Ayal

**Affiliations:** 1 Department of English Language and Literature, College of Social Sciences and Humanities, Wolidia University, Wolidia, Ethiopia; 2 Department of Public Health, College of Health Sciences, Woldia University, Woldia, Ethiopia; Bahir Dar University, ETHIOPIA

## Abstract

**Background:**

Reading comprehension is a critical skill for English as a foreign language learner. However, many Ethiopian secondary school students have been having trouble in reading comprehension, where English is a medium of instruction. Therefore, this study examined the impact of short stories on secondary school students’ reading comprehension and assessed their attitude towards short stories.

**Methods:**

A quasi-experimental design was employed. The participants were two sections of 9^th^ graders (n = 120) that were randomly assigned to experimental (n = 60), and control (n = 60) groups. The data were collected through a pre-and post-intervention reading comprehension test and an interview. Quantitative data were analyzed using independent and paired samples t-tests with effect sizes reported to strengthen statistical interpretation. Qualitative data were collected through interview with seven experimental group participants and analyzed thematically.

**Results:**

Results showed no significant difference between groups at pretest in their reading comprehension skills (t = 0.32, df = 118, p = 0.75). The post-test results showed that the experimental group (m = 10.38, sd = 2.63) significantly outperformed the control group (m = 6.72, sd = 2.57, p = 0.001). Within- groups analysis confirmed that the control group showed no significant change (p = 0.57). However, the experimental group showed significant change in reading comprehension test performance (p = 0.01). Interview findings displayed that short stories improved reading comprehension skills and fostered a positive attitude toward reading.

**Conclusion:**

The integration of short stories into English foreign language reading instruction significantly enhanced students’ reading comprehension skills. It also increased students’ positive attitudes in the reading texts. Therefore, it is recommended that English foreign language teachers should supplement reading skills instruction with culturally relevant short stories, and curriculum developers should incorporate more short stories in grade nine English textbooks to develop students’ reading comprehension skills.

## Introduction

The Ethiopian education and training policy emphasizes the development of the four language skills to enhance students’ communicative competence. Grammar and vocabulary are combined into the practices of these skills [1]. In foreign language learning, reading is recognized as a foundational skill that supports academic success and cognitive development across all secondary school levels. In contexts where English serves as the medium of instruction, strong reading comprehension is critical for students’ achievement in secondary education [2].

Reading comprehension is a key determinant of academic achievement, yet many Ethiopian students face persistent challenges in this area. Using short stories as a resource offers authentic literary texts that can stimulate interest, motivation, and deeper understanding. Their narrative structure, and culture relevance make them particularly, suitable for secondary school learners [3].

At the secondary school level in the Ethiopian context, where English is used as a medium of instruction, strong reading comprehension is essential for students’ future academic success in diverse fields of study. Good reading comprehension performance enhances achievement in higher education, where students encounter complex texts in different disciplines. To foster this skill, scholars have suggested integrating literary texts into language classrooms, as they are authentic, motivating, and capable of raising students’ interest in reading [4]. Therefore, short stories in particular, may provide a practical means of improving reading comprehension.

Despite the recognized benefits of reading comprehension, many students in Ethiopia find reading in English a challenging task to understand texts. They often lack effective strategies, which make reading discouraging and unproductive. Previous local studies have mainly employed descriptive designs and reported findings without testing the instructional effectiveness of literary texts through controlled interventions. Furthermore, teachers often perceived literary texts as difficult for secondary school students, limiting their classroom use. They often lack effective strategies of reading, which make reading a tough, unkind, and unproductive process. Readers will often be reluctant to read in the target language [5].

In addition, a study on grade ten students revealed that most of the literary texts in the textbook lacked sufficient literary text-based activity to provide learners with the opportunity to use numerous linguistic structures and registers for developing their language use. The distribution of literary genres was also quite unbalanced [6]. Another study focusing on grade 12 highlighted that both teachers and students had limited knowledge and low interest in integrating literary texts with language skills [7].

From classroom observation, the researchers noted that lots of EFL teachers perceived literary texts as difficult for secondary school students. Some English foreign language teachers also reported challenges in teaching literary texts due to in sufficient training in how o use them effectively in EFL classes. Nevertheless, this study advocates for the integration of literary texts, especially short stories into reading comprehension classrooms, emphasizing that teachers should consider learners’ needs, motivation, interests, cultural backgrounds, and language level. Doing so, they enhance students’ motivation or interest to understand the given reading comprehension texts and reading comprehension skills.

The study is based upon schema theory, which states that when reading, students comprehend by relating prior knowledge to what is being read. Short stories can facilitate this. Also, constructivist learning theory further recognizes that learners play a role in constructing knowledge, and this is evident within a literary text. Models of reading comprehension also highlight motivation and interest as facilitative processes, and these are evident within a short story. Conceptually, short stories are treated as the independent variable, and reading comprehension as the dependent variable, with motivation and interest as mediating variables. This framework illustrates how short stories can enhance students; reading comprehension skills by activating prior knowledge, fostering engagement, and supporting meaning-making.

Although international studies and theoretical perspectives highlight the benefits of short stories, empirical evidence from the Ethiopian context remains scarce. This study fills a clear gap by systematically testing the instructional effectiveness of short stories through a controlled intervention, rather than relying on descriptive accounts. It contributes theoretical significance by applying schema and constructivist learning theories to the Ethiopian context, pedagogical significance by offering evidence- based strategies for integrating short stories into EFL classrooms, and practical significance by addressing students’ attitudes. Therefore, this study investigates if short stories can facilitate reading comprehension skills and examines students’ attitudes toward reading short stories among grade nine secondary school students.

### Research objectives, questions and hypotheses

The objectives of this study are:

- To investigate the effect of short stories on secondary school students’ reading comprehension skills.
- To assess the attitude of secondary school students towards learning reading through short stories.

### Research questions

- Does the use of short stories significantly enhance the reading comprehension skills of secondary school students compared to the conventional instruction?
- What attitudes do secondary school students hold toward learning reading through short stories?

### Hypotheses

- The use of short stories will significantly enhance the reading comprehension level of the students compared to traditional methods.
- Students who are exposed to short stories will show more positive attitudes to reading in English.

## Methods

### Research design, setting and period

This study aimed to examine the effect of short stories on secondary school students’ reading comprehension, and assess students’ attitudes toward learning to read through short stories in EFL reading classes. Thus, a mixed method, quasi-experimental design was employed to explore cause-and-effect relationships between short story use and reading comprehension outcomes. The study was conducted at Wad Secondary School, located in Dembecha, Northwest Ethiopia. The school is a government run, and enrolls students from different socio-economic backgrounds. Recruitment of participants started on 1/02/2022 and ended on 3/02/2022. The intervention lasted for three months, with two sessions per week. Each took 40 minutes from February to April. During this period, the experimental group received instruction using four short stories, accompanied by pre-reading, while-reading, and post reading activities. The short stories, which are entitled “Serve her right,” “the Mother,” “Merkato,” and “Addis with Love,” were selected from grade 9 pupils’ reader. Selection criteria included students’ cultural background, language level, and interest. The control group followed the regular textbook contents. This time was chosen to offer participants enough time to engage with short stories, and for the researchers to observe measurable changes on students’ reading comprehension. Both the experimental and control groups were taught by the same teacher trained in advance to ensure consistency.

### Participants of the study and sampling techniques

**Fig 1:**
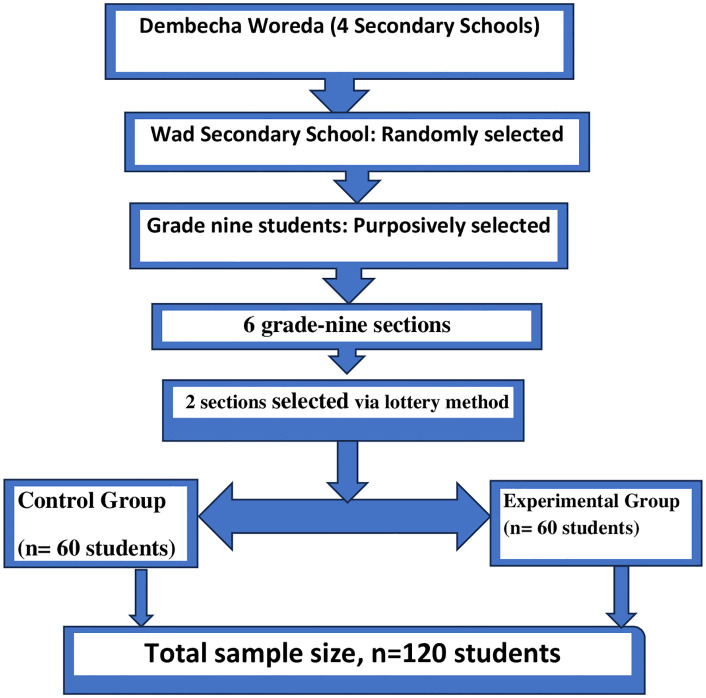
Sampling procedures for assessing short stories’ impact on secondary students’ reading comprehension skills in Dembecha Town.

Participants of the study were grade 9 students of Wad Secondary School in Dembecha Woreda, Northwest Ethiopia. The grade label was purposively selected because it marks a critical transition to secondary education, where students often face challenges in reading comprehension and motivation. The researcher had observed these difficulties during prior teaching and community service activities. Among the four secondary schools in Dembecha woreda, Wad Secondary School was selected using the simple random sampling method to ensure equal chances of selection of each school and minimize selection bias. Within the selected school, there were a total of six grade nine sections. Two sections were selected following a lottery system, with one assigned as the experimental and the other as the control group. Randomization occurred at the section level rather than the individual level to minimize crossover effects, since students within a section share classes and teachers. The total sample size was 120 (60 students per each group). A power analysis determined that at least 102 participants were required for independent sample t-tests (α = 0.05, power = 0.80, medium effect size, Cohen’s d = 0.5). The selected samples of 120 exceeded this threshold, ensuring adequate power to detect meaningful differences between groups. The relatively balanced group sizes also ensured comparability and reduced the risk of unequal variance between groups (**Fig 1**).

### Data gathering instruments

Reading comprehension tests and semi-structured interviews were used as the main instruments for data gathering. The reading comprehension pre- and post t-tests, each consisted of 20 multiple-choice items designed to assess the four sub- skills---vocabulary, inference, reference, and main idea comprehension. The deliberate choice of 20 items was made considering the test could be completed within a standard 40-minute class period without causing fatigue. This length also allowed adequate sampling of sub-skills while maintaining reliability. Items were developed based on the grade 9 English curriculum and reviewed for content validity by three anonymous subject- matter experts. Content validity was ensured through expert, and pilot testing was conducted in another school to refine item clarity. The reliability was determined using Cronbach’s alpha, which yielded coefficient of 0.82, indicating strong internal consistency. Moreover, semi-structured interviews were conducted with seven students from the experimental group. They explored the attitudes of students toward short stories, and their perceived effect on reading comprehension. Interview questions focused on interest, motivation, comparison with non-literary texts, and perceptions towards the grade 9 English textbook. Interview questions were also reviewed by three English language education experts to ensure alignment with study objectives.

### Data collection procedures

A pretest was administered to both groups prior to the intervention to establish baseline equivalence in reading proficiency. After the intervention had been completed, a posttest was administered to investigate the effect of the intervention on secondary school students’ reading comprehension skills. This allowed comparison of pre- and post- intervention performance to determine statistical significance. In addition, interviews were held with experimental group participants after the intervention to triangulate quantitative results, and provide deeper insight into students’ experiences. Standardized instructions and consistent classroom conditions were maintained to ensure reliability. All sessions were observed by the researchers to monitor fidelity of implementation.

### Methods of data analysis

The data from the test were analyzed statistically using an independent t-test to compare achievement of the experimental and control groups. Pre-test results were analyzed to confirm baseline equivalence (P > 0.05), ensuring that any subsequent differences could be attributed to the intervention. Furthermore, a paired samples t-test was used to examine within the group’s changes from pretest to posttest. Effect sizes (eta squared) were calculated to determine the magnitude of differences. Assumptions of parametric tests (normality, homogeneity of variance) were checked using Shapiro-Wilk and Levene’s tests, respectively. Then, the responses from the interviews were analyzed qualitatively, using thematic analysis. Codes were generated inductively from participants’ responses, grouped into themes, and reviewed for consistency. Peer checking was conducted to enhance reliability, and themes were compared against quantitative findings to ensure triangulation.

### Ethical consideration

Ethical approval for this study was obtained from the Institutional Review Board (IRB) of Woldia University. An official letter of permission was obtained from the Research and Community Service corporate Director of the social sciences and humanities college; furthermore, oral permission was obtained from the participating school director prior to data collection.

Because the participants were under the age of 18, written informed consent was obtained from the participants’ parents, and the participants were provided with age-appropriate information about the aim of the study, the procedures of the study, their voluntary participation, and the right to withdraw at any time without any penalty.

In addition, verbal assent was obtained from students to complement to parental written consent, given in the structured school setting. The verbal assent was documented by the researcher through written records recording the date, setting, grade level, and number of assenting students, and witnessed by the selected school director and the classroom teacher to ensure transparency and ethical integrity. This dual process of parental consent and student assent aligns with international ethical standards for research involving minor.

All data gained from the participants were used only for research purposes. Moreover, participant anonymity and confidentiality were strictly maintained in all the reports. Participation in the study did not provide financial or material compensation; however, participants were acknowledged for their contribution. To ensure fairness, the intervention was applied to the control group after the completion of the experimental phase and all necessary data had been collected from the experimental group to make sure that no group was disadvantaged and to maintain equal access to potentially beneficial pedagogy.

## Results

As Table 1 shows, Leven’s test confirmed equal variances (F = .161, P = .669, P > 0.05). The independent sample t-test confirmed no significant difference between the control group and experimental group pretest result (t = .32, df = 118, p = .75). This indicates that the two groups were statistically equivalent at baseline. The experimental group (n = 60) scored (M = 6.33, SD = 2.36), while the control group (n = 60) scored (M = 6.47, SD = 2.19). The mean difference (0.12) was very small (eta squared = 0.001). Thus, baseline equivalence was established, ensuring that post- intervention differences could be attributed to the treatment. In the independent samples t-test, it can be seen that the difference between the experimental group and the control group students was a non-significant one (t = 0.32, df = 118, p > 0.05) (**Table 1**).

**Table 1:** Pretest results of experimental and control groups.

Time	Leven’s test T-test for equality of mean								
	F	Sig	Group	No	Mean	SD	T	Df	Sig(2-tailed)
Pre-test	.161	.669	Experimental	60	6.33	2.36	.32	118	.75
			Control	60	6.45	2.19			

Leven’s test indicated unequal variances, (F = .321, P = .012, P < 0.05). The independent samples t-test revealed a significant mean difference between the control and experimental groups (t = 7.31, df = 118, p = 0.001, which is < 0.05). The experimental group (n = 60) achieved a score of (m = 10.38, SD = 2.63); on the other hand, the control group (n = 60) scored (m = 6.72, SD = 2.57). The mean difference (3.66) was large, with an effect size of (eta squared = .311), indicating a substantial practical impact. This confirms that the experimental group significantly outperformed the control group in reading comprehension after the intervention. Therefore, it is understood that teaching of reading through the use of short stories can result in student gains in reading in a foreign language (**Table 2**).

**Table 2:** Post-test results of experimental and control groups.

Time	Leven’s test (experimental control group)		t-test for equality of mean						
			Group	No	Mean	SD	T	Df	Sig(2Sig(2-tailed-tailed
	F	Sig							
Post-test	.321	0.012	Experimental	60	10.38	2.63	7.31	118	.0 001
			control	60	6.72	2.57			

The paired sample t-test indicated that there was a significant mean difference between the experimental group’s pretest result and posttest result. The experimental group’s mean increased from 6.33(sd = 2.36) at pretest to 10.38 (sd = 2.56) at posttest, yielding a mean difference of 4.05. This difference was statistically significant (t = 17, df = 59, p < 0.01). This shows that short stories had a strong positive impact on students’ reading comprehension achievement (**Table 3**).

**Table 3:** Within-Group comparison: Experimental group’s reading comprehension performance over Time.

Group	Number	Mean	Sd	T	Df	Sig(2-tailed)
Experimental Pre-test	60	6.33	2.36	17	59	.001**
Experimental Post-test	60	10.38	2.56			

** mean difference was significant at P < 0.01

As indicated in Table 4, the mean scores of the control group’s results of the posttest and the pretest were 6.47 and 6.72, respectively. A 2-tailed paired samples t-test for mean difference showed that the mean gain between the post- and pretest mean scores was 0.25 (df = 59). This mean gain between the pre- and posttest mean scores of the control group was not statistically significant at the 0.05 level of significance since p = 0.57 > 0.05. This indicates that non-literary texts didn’t produce measurable improvement in students’ reading comprehension (Table 4).

**Table 4:** Within-Group comparison: control group’s reading comprehension performance over time.

Group	Number	Mean	SD	T	Df	Sig(2-tailed)
Control pre-test	60	6.47	2.19	.574	59	0.57
Control Post-test	60	6.72	2.57			

Sig. P > 0.05

### Data analysis of the interview

After the intervention had been completed, semi-structured interviews were conducted with seven participants from the experimental group, using purposive maximum variation sampling method. Participants were selected to reflect variation in reading performance levels on post-test scores, ensuring diversity of perspectives. All interviews were audio- recorded, transcribed verbatim, and conducted in Amharic. Each interview lasted approximately 25–30 minutes. The qualitative data were analyzed using thematic analysis following Braun and Clarke’s six-phase framework (familiarization, coding, theme generation, review, definition and reporting). Coding was conducted by two independent coders, achieving inter-coder reliability Cohen’s κ = 0.78), indicating substantial agreement. Discrepancies were resolved through discussion and consensus. Peer debriefing was conducted to enhance analytic rigor and credibility.

Thematic saturation was used to guide sample adequacy. No new codes or themes emerged after the fourth interview, and the fifth interview confirmed redundancy. Therefore, saturation was achieved, supporting the adequacy of the sample size (n = 7). The analysis generated the following four analytically distinct, but related themes, with participants contributing to multiple themes.

### Initial lack of exposure and evolving reading interest

All participants (7/7) reported minimal exposure of learning to read through short stories and little interest in literary texts, as they did not read any short stories before the intervention. Short stories were not part of their regular reading experience, contributing to low engagement with reading tasks. However, participants consistently described a shift in attitude following the intervention, expressing increased interest in reading both inside and outside the classroom.

One respondent reflected, “I had said nothing about short stories before I learned to read through those short stories, but now I have a good feeling about reading short stories inside and outside the classroom.”

This finding suggests that exposure to short stories transformed students’ reading attitudes, cultivating both reading interest and the habit of reading and beyond the classroom.

### Narrative relevance and authenticity as drivers of engagement

Five participants (5/7) emphasized the engaging nature of short stories compared to actual texts due to the narrative nature and relatability. Participants noted that short stories reflected real-life situations, cultural contexts and meaningful language use, which increased their engagement to read.

For example, participants explained, “The short stories we read are more interesting than what we had read in the actual classroom since they told us different notions and functions of our real life.”

This implies that the students were really interested because of the narrative nature of short stories, and they were very motivated as they involved themselves within the situations or events described in the short story. In contrast, non-literary reading texts, filled with factual information, and lacked this motivating structure.

### Comparison with non-literary texts

Three participants explicitly contrasted short stories with actual foreign language text, describing the latter as fact-based, less engaging and similar to other academic subjects. For instance, respondents responded, “The reading texts used in the actual classroom felt like other natural science content and didn’t motivate them to read well, and reading texts used in the actual classroom tells us just general truths and facts, whereas short stories inform us about things-- culture, language, etc.- in our day-to-day situation.” This highlights literary texts (short stories) were perceived as interesting reading materials, which motivates students to read more as they relate with their real-life situations. This underscores the selected short stories for reading purposes should be rich in context and meaningful to enhance students’ reading comprehension.

### Perceived improvement in reading comprehension and confidence

All participants (7/7) reported that short stories improved their reading comprehension skills and confidence in answering comprehension questions. Participants described greater ease in interpreting meaning, making inferences, and engaging with reading tasks.

One respondent explained, “Before learning to read through short stories, we couldn’t understand the text and answer comprehension questions confidently, but now we can do better than previously.”

This showed that short stories can improve performance and confidence in the reading comprehension skills of students, as they have the power of initiating the feelings of students.

### Instructional and curriculum implications

All participants (7/7) highlighted the role of teachers and curriculum in facilitating access to engaging reading materials. Participants recommended that English foreign language teachers supplement textbooks with short stories and more literary texts be incorporated into the grade English curriculum.

For instance, participants suggested, “Grade nine students’ English textbook should contain many short stories for reading purposes because they assist us to read and understand better than before; however, there are literary texts, very few in number compared with non-literary reading texts.”

This showed that students understood the scarcity of short stories, or literary texts in the curriculum, and advocated for greater inclusion, linking short stories directly to improved reading comprehension skills.

Overall, the qualitative analysis indicates that short stories enhance students’ engagement, motivation and perceived reading comprehension skills, primarily through narrative relevance and contextualized language use. While participants consistently reported improved comprehension and confidence, these findings should be interpreted as perceptual evidence that complements the statistically verified improvements in reading performance.

The integration of qualitative and quantitative results supports an explanatory mixed-methods interpretation, where interview data help explain the mechanisms (engagement, motivation, schema activation) underlying observed gains in reading comprehension. Thus, interviews corporate the direction of quantitative findings by explaining why improvement occurred, particularly through engagement and contextual relevance, but they do not independently establish causal effects. The following table summarizes the distribution of themes, participant representation and illustrative quotations (**Table 5**).

**Table 5:** Thematic mapping of interview findings (n = 7).

Themes	Participants (n = 7)	Representative quotes	Link to research questions
Initial lack of exposure and evolving reading interest	7/7	“I had said nothing about short stories before I learned to read through those short stories, but now I have a good feeling about reading short stories inside and outside the classroom.”	Attitudes
Narrative relevance and authenticity as drivers of engagement	5/7	“The short stories we read are more interesting than what we had read in the actual classroom since they told us different notions and functions of our real life.”	Attitudes
Comparison with non-literary texts	3/7	“They are like science subjects…”	Attitudes
Perceived improvement in reading comprehension and confidence	7/7	“Before learning to read through short stories, we couldn’t understand the text and answer comprehension questions confidently, but now we can do better than previously.”	Reading comprehension skills
Instructional and curriculum implications	7/7	“Grade nine students’ English textbook should contain many short stories for reading purposes because they assist us to read and understand better than before; however, there are literary texts, very few in number compared with non-literary reading texts.”	Pedagogy

## Discussion

This study addressed two research questions:(1) does the use of short stories enhances students’ reading comprehension skills, and (2) what attitudes students hold toward learning reading through short stories. Regarding the first research question, the quantitative results of the experimental group’s posttest mean score (M = 10.38, SD = 2.63), which was treated with short stories, significantly outperformed the control group (M = 6.72, SD = 2.63), with a large effect sizes (η² = .311).In addition, within-group analysis confirmed that the experimental group’s results significantly improved from pre- to post- intervention program (mean difference = 4.05 < 0.001). These results show that the intervention with short stories had a substantial effect on students’ reading comprehension, while the control group showed no significant change.

With respect to the second research question, the qualitative findings showed that students held positive attitude toward short stories, reporting increased interest, engagement, and motivation in reading. Participants also perceived improvements in their reading comprehension skills and confidence.

The qualitative findings supported the quantitative results by providing explanatory insights into how short stories facilitated reading comprehension skills. The students described short stories as interesting reading text that motivated them to read more and relate it to their own real experience, in contrast to non-literary texts. They also emphasized that short stories enhanced their reading comprehension skills, confidence, language use, and daily life. The convergence of quantitative and qualitative data strengthens the validity of the findings and underscores the pedagogical value of short stories.

Theoretically, the results align with schema theory, which posits that readers understand texts more effectively when the content activates their prior knowledge and emotional frameworks—both features strongly embedded in narrative texts. Short stories, because of their usual message and character-driven storyline, have been shown to help students use new information and relate new knowledge to ideas that they already know. By connecting familiar cultural contexts with new language input, short stories bridge the gap between prior knowledge and reading comprehension [8].

Reader-response theory further highlights the importance of emotional engagement in meaning making. Students’ positive comments about short stories reflect Rosenblatt’s notion of transactional reading, where interaction with text fosters deeper comprehension. This emotional involvement explains why short stories were perceived as more motivating than factual texts [9].

Similarly, principles of second language acquisition, particularly Krashen’s Input Hypothesis, supports the role of understandable and engaging language learning resources that contribute much to language acquisition. Thus, short stories serve as both linguistic input and motivational stimulus, fulfilling dual roles in English foreign language learning [10].

These results are also consistent with international empirical studies, which show that short stories boost increased engagement, imagination, and critical thinking, which sequentially develop comprehension outcomes [11]. Studies in Hong Kong have showed similar findings---when short stories were integrated into reading instruction, the students’ reading achievement was improved [12]. In addition, there is a study that indicated though literary texts are more challenging than textbooks, they can enhance the joy of reading, as they are authentic or “real” literature [13].

In the same vein, the use of literary works in a language classroom can facilitate the learning of the target language [14]. Furthermore, learners also profit from literary texts. What they read gives them the opportunity to come up with their own insights and helps them to use the language in a more imaginative way. They become more creative since they are faced with their own point of view [15]. While some other scholars emphasized that literary texts may be linguistically difficult for English foreign language learners, the current study reveals that appropriately selected short stories can be effective for the improvement of reading comprehension skills.

Both theoretical perspectives and empirical studies outside Ethiopia support the effectiveness of using short stories as an instructional strategy. The significant improvement in the experimental group suggests that short stories offer enriched input, motivational benefits, and cognitive engagement beyond what is typically provided through traditional textbook passages. The alignment of quantitative and qualitative results underscores the strength of the current findings and highlights the value of integrating literary narratives into Grade 9 reading instruction.

Though much of the existing literature backs these decisions, some scholars have raised concerns about using literary texts in EFL settings. One noticed that fictional writings might be too hard for EFL students, hindering comprehension [16]. Though the Grade 9 students in this study answered positively and completed meaningfully higher comprehension grades, verifying that appropriately selected short stories improve understanding instead of hindering it. The other study showed that EFL/ESL teachers always experience difficulties while using literary texts as reading teaching material because of inappropriate training and skill in approaches of teaching [17]. In addition, there is local research that claimed that literary texts are hardly used in Ethiopian English foreign language teaching contexts since EFL teachers get it hard linguistically and take much time to use it for teaching reading purposes [18]. These findings might be right in some aspects, but the current study shows that with proper teaching support, using short stories is effective in enhancing students’ reading comprehension and interest.

Taken together, the findings demonstrate that short stories can simultaneously enhance reading comprehension and cultivate positive attitudes toward reading. The findings have several important implications for English foreign language teaching practice by applying Schema learning theory to the Ethiopian context, pedagogical significance by offering evidence- based strategies for integrating short stories into English foreign language classrooms, and practical significance by addressing students’ attitudes. First, English language teachers should consider integrating short stories into reading instruction to enhance both reading comprehension skills and motivation. Second, teachers should carefully select texts that are culturally relevant and linguistically appropriate to maximize effectiveness. Third, short stories can be used alongside pre,while and post-reading activities to support comprehension. In addition, the findings suggest a need for curriculum development. Students consistently reported that their textbooks contain insufficient literary texts. Incorporating more short stories into the grade 9 English curriculum could improve both engagement and learning outcomes.

### Limitations and future research

This study was conducted in a single secondary school with a relatively small sample size (n = 120), which may limit generalizability. The intervention lasted only three months, and longer-term effects were not examined. In addition, the qualitative data were collected from seven purposively selected participants (n = 7), which may not capture all possible experiences although thematic saturation was achieved. Future research should replicate this study across multiple schools, extend the intervention period with larger and more diverse samples to strengthen the generalizability of the findings.

## Conclusion

The result of this study provides strong evidence that integrating short stories into English foreign language reading instruction significantly improved secondary school students’ reading comprehension and increased positive attitudes towards reading. These findings suggest that short stories provide authentic, culturally relevant input that fosters both cognitive and affective dimensions of reading. It is recommended that the chance of choosing reading texts should have been given to both the EFL teachers and students. Besides, teachers should supplement instruction with interesting short stories that could go in line with the English language performance, cultural background and linguistic level of students, and that curriculum developers should incorporate more short stories into the grade 9 English textbook to support students’ reading and comprehension skills and attitudes.

## Supporting information

S1 Appendix Pre- test questions used for grade 9students, contains reading comprehension questions adapted from the Grade 9 English textbook. (DOCX)

S2 Appendix Post-test questions used for Grade 9 students, contains reading comprehension questions adapted from Tottman (1972). (DOCX)

S3 Appendix Students’ interview guide, includes semi-structured questions on attitudes toward short stories and reading comprehension. (DOCX)

S1 Table Pre-test results of the two groups. (DOCX)

S2 Table Results of post-test for the two groups computed using an independent t-test. (DOCX)
