# Supplementary material for: The effect of short stories on secondary school students’ reading comprehension skills and attitudes in Northwest Ethiopia
Source: PLoS One. 2026 Jun 1;21(6):e0350250. doi: 10.1371/journal.pone.0350250 (PMC13225352; doi:10.1371/journal.pone.0350250)
Supplement: S1 Appendix — (DOCX) [file pone.0350250.s001.docx]

**S1 Appendix. Pre-test questions used for grade 9 students, contains reading comprehension questions adapted from Grade 9 English textbook**

**Wolidia University**

**College Of Social Science and Humanities**

**Department Of English Language and Literature**

**Reading comprehension questions prepared for grade 9^th^ students of Wad Secondary school**

**General direction**: For items 1-20, read the passage carefully. And then, choose the best answer for each question according to the information given in the passage. (Time allowed: 50 minutes**)**

1. Ethiopia remains one of Africa’s most traditional societies, even when it comes to the law. Although the country has great ethnic, religious, cultural diversity, and attitudes towards woman’s rights are relatively homogenous in rural societies, where harmful traditional practices, which particularly affect females, are the norm.

2. The 1960 civil law does not secure rights for women. It rather treats them as if they were children or disabled people. Child prostitution is on the increase, especially in major urban centers of the country. Girls as young as age 11 are recruited to work in houses of prostitution, where they are kept ignorant of the risks of HIV infection. In 1996, a National Steering Committee against Sexual Exploitation of children was formed. In October of the same year, the committee reported that child prostitution continued to be a problem. There have been many reports of the large-scale employment of children, especially underage girls, as hotel workers, barmaids, and prostitutes in towns of recreation and truck stops south of Addis Ababa. Social workers note that girls are prized because their sexual partners believe that they are free of sexually transmitted diseases.

3. Other harmful traditional practices surveyed by the National Committee on Traditional practices in Ethiopia included uvulectomy, milk-teeth extraction, early marriage, and marriage by abduction. The constitution defines the age of lawful marriage as 15 for females and 18 for males. Nevertheless, early childhood marriage is common in rural areas, with girls as young as 9 given in marriage. In the Afar region of the east, young girls continue to be married to much older men, but this traditional practice is coming under greater scrutiny and criticism because pregnancy at an early age often leads to obstetric fistulae, resulting in lifelong misery. The maternal mortality rate is extremely high due to, in part, food taboos for pregnant women, early marriage, and birth complications related to female genital mutilation.

4. Although illegal, the abduction of women and girls as a form of marriage is still widely practiced in the Oromiya and the Southern Nations Nationalities and Peoples Region regional states. Forced sexual relationships often accompany most marriages by abduction, and a woman is often abused physically during the abduction. Experience shows us that abduction has led to conflicts between families’ communities and ethnic groups.

5. Critical issues affecting women and children include unfair penalties for rapes, and domestic violence. However, regardless of changes to the penal code, traditional and cultural views often exist in civil and criminal law, and in practice women do not enjoy equal status with men.

6. Societal abuses of young girls continue to be a problem. Reliable statistical data shows that 72.7% of the female population undergo female genital mutilation (FGM) or genital cutting. Clitoridectomies typically are performed 7 days after birth. Infibulation, the most extreme and dangerous form of FGM, is performed at any time between the age of 8 and the onset of puberty. This practice is widely condemned by international health experts as damaging to both physical and psychological health. The law does not specifically prohibit FGM, although it is discouraged officially, and the government has been very supportive of the National Committee. The government also is working to discourage the practice of FGM through education in public schools. On the other hand, the National Committee and other concerned organizations say that cultural practice encourages women to want to undergo one of the four circumcision procedures. It is often associated with positive attributes, such as gaining respect within the village and becoming a woman. Most importantly, girls who have not undergone one of these procedures are considered more likely to be promiscuous and, therefore, unworthy of marriage. The belief also exists that external female genitals are unclean. Some use religion as the basis for their justification in performing these procedures, despite the fact that they are not required by either the Quran or the Bible.

(**Adapted from grade 9 English students’ textbook)**

**Part 1: Scanning and skimming questions**

**For items 1-10, read the passage, and then choose the best answer for each question.**

1. The main purpose of paragraph 1 is to ________

A. Explains harmful traditional practices on women.

B. Remained attitudes of the society are homogeneous.

C. Explains the women’s rights are properly implemented.

D. All are possible purposes of the paragraph.

2. According to the passage, which is correct?

A. Marriage by abduction is a useful traditional practice.

B. Women are properly treated during the abduction.

C. The 1960 civil law secures rights for women.

D. Marriage by abduction leads to conflict between families.

3. What does paragraph 2 explain? It…………

A. Explain how the 1960 civil law secures rights for women.

B. Explain about other traditional practices surveyed by the national committee.

C. Explain how abduction of women and girls is practiced.

D. Explain how maternal mortality is caused by birth complications.

4. Which of the following phenomena is not a harmful traditional practice according to the passage?

A. Milk-teeth extraction B. Early marriage C. Child prostitution D. Lawful marriage

5. Which one of the following might be the purpose of the passage?

A. Warning B. Entertaining C. Advising D. Apologizing

6. What is the central idea of the passage is?

A. Childhood marriage and abduction are harmful traditional practice.

B. Civil law secures rights for women.

C. Abduction of women and girls is widely controlled.

D. The international health experts condemned harmful traditional practices.

7. According to the passage, the author wants to show….

A. Cultural diversity

B. Nations and Nationalities of the Country.

C. Attitudes of rural societies.

D. Harmful traditional practices are widely practiced.

8. Which one of the following is not true about paragraph 3?

A. Harmful traditional practices have been controlled by the national committee.

B. The constitution defines lawful marriage for females and males.

C. Young girls are forced to marry older men.

D. Lifelong misery is caused by an early-age pregnancy.

9. What is the purpose of paragraph 4?

A. Inform about the effect of abduction of women.

B. Inform how abduction of women can be managed.

C. Explain how women abduction can easily be avoided.

D. Inform how women abduction is widely practiced.

1O. Which one of the paragraphs talks about genital cutting?

A.3 B.4 C.6 D.2

**Part 2: Reference questions**

**For items 11-14, read the passage, and then choose the best answer for each question.**

11. What does the expression” **the same year”** in paragraph 2 indicate?

A. The 1960 B. The 1996 C. Before 1960 D. After 1996

12. The pronoun “**it’** (paragraph 2) refers to………….

A. Rights for women B. Child prostitution C. The 1960 civil law D. Urban center

13. What does the word” **them”** in paragraph 2 refer to?

A. The women B. Rights of women C. Disabled people D. National steering committee.

14. The pronoun “**their”** in paragraph 2 refers to ………

A. Social workers B. Girls C. Hotel workers D. Prostitutes

**Part 3: Vocabulary items**

**For items 15-20, read the passage and then choose the best answer for each question.**

15. The word “**homogenous”** in paragraph 1 means what?

A. Different B. Similar C. Varied D. opposite

16. What does the word “**abduction”** in paragraph 1 mean?

A. Illegal action B. Interaction C. Mortality D. Discussion

17. The word “**prized”** in paragraph 2 means?

A. Selected B. Highly valued C. Ignored D. Succeeded

18. What does the word “**condemned”** in paragraph 6 mean?

A. Suited B. Disproved C. Accepted D. Denied

19. What does the word “**attributes** “in paragraph 6 mean?

A. Qualified behavior B. Unsuited conduct C. Gained result D. Unwanted quality

20. What does the phrase …” **the onset of puberty”** as used in paragraph 6, mean?

A. At the end of puberty.

B. At the beginning of puberty.

C. At the middle of puberty.

D. At any time of puberty.
