# Supplementary material for: The effect of short stories on secondary school students’ reading comprehension skills and attitudes in Northwest Ethiopia
Source: PLoS One. 2026 Jun 1;21(6):e0350250. doi: 10.1371/journal.pone.0350250 (PMC13225352; doi:10.1371/journal.pone.0350250)
Supplement: S2 Appendix — (DOCX) [file pone.0350250.s002.docx]

**S2 Appendix. Post-test questions used for grade 9 students, contains reading comprehension questions adapted from Tottman (1972)**

**Wolidia University**

**College of Social Sciences and Humanities**

**Department of English Language and Literature**

**Reading comprehension questions prepared for grade 9^th^ students of Wad Secondary School.**

**General direction**: For items 1-20, read the passage carefully. Then, choose the best answer for each question according to the information given in the passage. (Time allowed: 50 minutes)

The human body is made up mainly of bones, muscle, and fat. Some 639 different muscles account for about 45% of the body weight. Each of these muscles has distinct and measurable qualities that are of interest to us:

1. It can produce force which can be measured as strength of muscle.

2. It can store energy which permits it to work for extended periods of time independently

of circulation-this is generally referred to as muscular endurance.

3. It can shorten at various rates. This is called muscular contraction.

4. It can be stretched and will recoil. This is called the elasticity of the muscle.

The combination of these four qualities of muscle is referred to as muscular power. If muscles are to function efficiently, they must be continually supplied with energy fuel. This is accomplished by the blood, which carries the energy from the lungs and digestive system to the muscles. The blood is forced through the blood vessels by the heart. The combined capacity to supply energy fuels to the working muscles is called organic power.

The capacity and efficiency with which your body can function depends on the degree of development of both your muscular and organic power through regular exercise. However, the level to which you can develop the powers is influenced by such factors as the type of body you have, the food you eat, the presence or absence of disease, rest, and sleep. You are physically fit only when you have adequately developed your muscular and organic power to perform with the highest possible efficiency.

Heredity and health determine the top limits to which your physical capacity can be developed. This is known as your potential physical capacity. This potential capacity varies from individual to individual. Most of us, for example, could train for a lifetime and never come close to running a four-minute mile simply because we were not built for it. The top level at which you can perform physically right now is called your acquired capacity because it has been acquired or developed through physical activity in your daily routines.

Your body, like a car, functions most efficiently well below its acquired capacity. A car, for example, driven at its top speed of say,110 miles per hour, uses more petrol per mile than when it is driven around 50-60 miles per hour, which is well below its capacity. Your body functions in the same way, in that the ratio of work performed to energy expended is better when it functions well below acquired capacity.

You can avoid wastage of energy by acquiring a level of physical capacity well above the level required to perform your normal daily tasks. This can be accomplished by supplementing your daily physical activity with a balanced exercise program performed regularly. Your capacity increases as you progressively increase the load on your muscular and organic systems. Exercise will increase physical endurance and stamina, thus providing a greater reserve of energy for leisure-time activities.

Just as a balanced diet must be composed of a sufficient quantity of the proper kinds of food to ensure that nutritional requirements are adequately met, so should a balanced physical activity program be composed of a sufficient quantity of the proper kind of physical activity so that all the important parts of the body are adequately exercised. The parts of the body that require special attentions are the muscles of the shoulders and arms, abdomen and back, legs, and the heart, lungs and blood vessels.

No single sport provides a truly balanced development for all parts of the body. This can only be acquired by regular participation in a number of carefully selected sports. Such participation, however, is not possible for the average person for a number of reasons--availability of play opportunity, time expense. The most practical physical fitness scheme for most of us is participation in one or two sports supplemented by a balanced set of exercises.

**(Adopted from English Comprehension through Objective Tests (Tottman S., 1972).**

1. Muscular strength is measured by the muscles’

A. Power B. Control C. Stamina D. Pressure

2. Elasticity of muscles refers to the muscular ability to

A. Expand and contract C. Gradually decrease in size

B. Become lighter and heavier D. Move in and out

3. Muscular power includes all of the following except

A. Strength of muscle C. Speed of contraction

B. Muscular endurance D. Independence of circulation

4. An expression to replace ‘recoil’ (line 8) could be

A. Spring forward C. Jump away

B. Spring back D. Pull up

5. This passage is mainly written for

A. Those that is anxious about their health

B. people with little knowledge of how the body works

C. Medical students

D. physical education instructors

6. Energy fuel is carried to the muscles by the

A. Digestive system B. Lungs C. Blood D. Heart

7.The effective functioning of the body can be improved by

A. strength of muscles C. muscular power

B. regular exercise D. organic power

8. Another way of expressing ‘highest possible efficiency’ (line 21) is-------

A. Maximum effectiveness C. organic power

B. best use of muscle D. optimum strength

9.‘Heredity’ (line22) refers to characteristics which we-------.

A. obtain through regular exercise

B. develop by the way we live

C. acquire from our parents

D. learn through experience

10. The passages states that it is possible for everyone to

A. obtain a perfect physique

B. develop his full physical capacity

C. be completely efficient

D. be the same as everybody else

11. According to the passage, one’s body is like a car (line 30) because it

A. performs best at maximum power

B. is best used with its physical limits

C. needs plenty of fuel

D. Goes best at very low speeds

12. Wastage of energy can be avoided by----------.

A. Conserving one’s strength

B. Always working hard

C. Performing one’s daily tasks well

D. Extending one’s physical capacity

13. The level of your physical capacity can be increased by-----------.

A. regular balanced exercise

B. good eating habits

C. rest and relaxation

D. controlled exercises

14. Leisure-time activities (line 43) are activities we engage in when we-----------.

A. Need to feel up our time

B. Don’t know what to do

C. Feel too tired to do

D. Are free to do what we wish

15. A’ balanced diet’ (line 44) must be composed of -------------.

A. the right foods in the correct amounts

B. plenty of protein foods

C. cooked meals served regularly

D. small amount of food regularly taken

16. To ensure that all parts of the body are ‘adequately exercised’ (line 48) we should—

-----------.

A. meet all nutritional requirements

B. take plenty of strenuous exercise

C. exercise according to a proper plan

D. go walking and swimming as much as possible

17. The average person can’t participate in a number of carefully selected sports (line 54) because he----------.

A. hasn’t the facility, money or time

B. is unable to master them all

C. hasn’t the physical endurance

D. would only exhaust himself

18. The ‘most practical’ (line 55) way to keep fit is to participate in -----------.

A. one sport demanding vigorous activity

B. several sports requiring stamina

C. as many sports as possible

D. some sports with carefully chosen exercise

19. The purpose of this passage is mainly to---------------.

A. present facts

B. describe the working of the body

C. Persuade the reader to keep fit.

D. instruct the reader

20. Another word which could replace the word ‘supplemented’ (line 56) is--------.

A. sustained

B. varied

C. completed

D. complemented
