# Supplementary material for: The effect of short stories on secondary school students’ reading comprehension skills and attitudes in Northwest Ethiopia
Source: PLoS One. 2026 Jun 1;21(6):e0350250. doi: 10.1371/journal.pone.0350250 (PMC13225352; doi:10.1371/journal.pone.0350250)
Supplement: S3 Appendix — (DOCX) [file pone.0350250.s003.docx]

**S3 Appendix. Students’ interview guide, includes semi-structured questions on attitudes toward short stories and reading comprehension**

**Wolidia University**

**Collage of Social Sciences and Humanities**

**Department of English Language and Literature**

Dear students,

I would like to inform you that all the information that you will give in this interview will be strictly kept confidential and will only be used for the purpose of this research. This interview will focus on your attitudes on improving reading comprehension skill after completing reading of the given short stories. If you don’t understand any of the questions, please feel free to ask or interrupt me during the interview.

1. What is your overall opinion of short stories for learning to read in this study?

2. Does reading short stories increase your reading interest than reading non-literary texts from your English for Ethiopia grade 9 textbook? If yes, why?

3. Do you think reading short stories helps to improve your reading comprehension skills? If yes, why?

4. What do you think of the current English for Ethiopia student text book for grade 9th in relation to the adequacy of short stories incorporated in the text book?

5. Is there anything else that you would like to say before we end the interview?

**Adopted from (Baba, 2008).**
