# Supplementary material for: The effect of short stories on secondary school students’ reading comprehension skills and attitudes in Northwest Ethiopia
Source: PLoS One. 2026 Jun 1;21(6):e0350250. doi: 10.1371/journal.pone.0350250 (PMC13225352; doi:10.1371/journal.pone.0350250)
Supplement: S1 Table — (DOCX) [file pone.0350250.s004.docx]

**S1 Table 1. pre-test results of the two groups**

| **Participants’**  **code** | **Results of the pre-test for the two sections** | |
| --- | --- | --- |
|  | **9^th^ B** | **9^th^ E** |
| 1 | 7 | 6 |
| 2 | 7 | 5 |
| 3 | 5 | 9 |
| 4 | 7 | 8 |
| 5 | 9 | 9 |
| 6 | 7 | 9 |
| 7 | 11 | 4 |
| 8 | 10 | 4 |
| 9 | 9 | 3 |
| 10 | 11 | 7 |
| 11 | 5 | 6 |
| 12 | 7 | 7 |
| 13 | 6 | 4 |
| 14 | 10 | 5 |
| 15 | 5 | 9 |
| 16 | 5 | 6 |
| 17 | 9 | 5 |
| 18 | 4 | 9 |
| 19 | 4 | 9 |
| 20 | 7 | 9 |
| 21 | 6 | 6 |
| 22 | 8 | 6 |
| 23 | 10 | 6 |
| 24 | 10 | 8 |
| 25 | 6 | 10 |
| 26 | 5 | 5 |
| 27 | 5 | 7 |
| 28 | 6 | 9 |
| 29 | 8 | 6 |
| 30 | 6 | 8 |
| 31 | 5 | 1 |
| 32 | 9 | 5 |
| 33 | 8 | 7 |
| 34 | 7 | 11 |
| 35 | 6 | 4 |
| 36 | 5 | 9 |
| 37 | 3 | 4 |
| 38 | 7 | 10 |
| 39 | 5 | 3 |
| 40 | 9 | 6 |
| 41 | 4 | 2 |
| 42 | 4 | 6 |
| 43 | 6 | 6 |
| 44 | 9 | 6 |
| 45 | 5 | 2 |
| 46 | 6 | 5 |
| 47 | 5 | 7 |
| 48 | 6 | 5 |
| 49 | 5 | 6 |
| 50 | 5 | 6 |
| 51 | 3 | 5 |
| 52 | 7 | 3 |
| 53 | 7 | 8 |
| 54 | 11 | 7 |
| 55 | 4 | 1 |
| 56 | 4 | 11 |
| 57 | 6 | 7 |
| 58 | 7 | 9 |
| 59 | 3 | 8 |
| 60 | 2 | 6 |
| mean | 6.47 | 6.33 |
| Standard Deviation | 2.19 | 2.36 |
| Mean difference | 0.14 | |
| T-value | 0.321 | |
