# Supplementary material for: The effect of short stories on secondary school students’ reading comprehension skills and attitudes in Northwest Ethiopia
Source: PLoS One. 2026 Jun 1;21(6):e0350250. doi: 10.1371/journal.pone.0350250 (PMC13225352; doi:10.1371/journal.pone.0350250)
Supplement: S2 Table — (DOCX) [file pone.0350250.s005.docx]

**S2 Table 2. Results of post-test for the two groups computed using an independent t-test**

| **Participants’**  **Code** | **Results of the post-test for the two groups** | |
| --- | --- | --- |
|  | **9^th^ E (Experimental group)** | **9^th^ B (Control group)** |
| 1 | 9 | 9 |
| 2 | 10 | 6 |
| 3 | 13 | 5 |
| 4 | 12 | 10 |
| 5 | 13 | 4 |
| 6 | 13 | 4 |
| 7 | 10 | 7 |
| 8 | 8 | 6 |
| 9 | 8 | 10 |
| 10 | 11 | 13 |
| 11 | 10 | 4 |
| 12 | 13 | 5 |
| 13 | 9 | 7 |
| 14 | 12 | 12 |
| 15 | 15 | 4 |
| 16 | 12 | 9 |
| 17 | 9 | 11 |
| 18 | 14 | 7 |
| 19 | 12 | 5 |
| 20 | 13 | 4 |
| 21 | 10 | 9 |
| 22 | 12 | 3 |
| 23 | 9 | 6 |
| 24 | 13 | 5 |
| 25 | 13 | 10 |
| 26 | 8 | 8 |
| 27 | 13 | 5 |
| 28 | 14 | 8 |
| 29 | 10 | 7 |
| 30 | 13 | 6 |
| 31 | 6 | 5 |
| 32 | 10 | 8 |
| 33 | 11 | 10 |
| 34 | 6 | 8 |
| 35 | 7 | 5 |
| 36 | 14 | 3 |
| 37 | 9 | 7 |
| 38 | 15 | 9 |
| 39 | 7 | 10 |
| 40 | 10 | 10 |
| 41 | 7 | 5 |
| 42 | 10 | 3 |
| 43 | 12 | 7 |
| 44 | 10 | 8 |
| 45 | 8 | 5 |
| 46 | 10 | 3 |
| 47 | 12 | 7 |
| 48 | 11 | 5 |
| 49 | 10 | 10 |
| 50 | 9 | 6 |
| 51 | 5 | 3 |
| 52 | 3 | 8 |
| 53 | 8 | 10 |
| 54 | 10 | 10 |
| 55 | 5 | 2 |
| 56 | 14 | 5 |
| 57 | 11 | 7 |
| 58 | 12 | 6 |
| 59 | 11 | 5 |
| 60 | 9 | 4 |
| mean | 10.38 | 6.8 |
| Standard Deviation | 2.56 | 2.65 |
| Mean difference | 3.58 | |
| T-value | 26.513 | |
